# The effect of cigarette and e-cigarette use on periodontal health: A cross-sectional study in Eastern Province, Saudi Arabia

**DOI:** 10.18332/tid/209573

**Published:** 2026-01-17

**Authors:** Eman A. Aljoghaiman, Ali Albrahim, Abdullah Aldarisi, Majid Alsafwani, Faisal Alhalal

**Affiliations:** 1 Department of Preventive Dental Sciences, College of Dentistry, Imam Abdulrahman Bin Faisal University, Dammam, Saudi Arabia; 2 College of Dentistry, Imam Abdulrahman Bin Faisal University, Dammam, Saudi Arabia

**Keywords:** e-cigarette, periodontal disease, oral health, smoking, nicotine

## Abstract

**INTRODUCTION:**

Periodontal disease is a prevalent inflammatory condition influenced by various risk factors, including tobacco use. With the rising popularity of electronic cigarettes (e-cigarettes), their potential impact on periodontal health warrants investigation. The aim was to assess the association between e-cigarette use and periodontal disease compared to traditional cigarette smokers and non-smokers in a Saudi adult population.

**METHODS:**

This cross-sectional study included 169 adults in the Eastern Province of Saudi Arabia. Participants completed questionnaires on demographics, tobacco use, and oral hygiene practices. Periodontal status was clinically assessed. Logistic regression was used to evaluate the association between tobacco type and periodontal disease, adjusting for confounders.

**RESULTS:**

Periodontal disease was diagnosed in 66.9% of participants. Tobacco use was reported by 88%, with 37.3% using e-cigarettes exclusively. Cigarette and mixed users had the highest prevalence of disease, followed by e-cigarette users, with non-smokers showing the lowest rates. Logistic regression revealed significantly increased odds of periodontal disease in cigarette smokers (adjusted odds ratio, AOR=16.31; 95% CI: 2.16–123.18; p=0.0071), and elevated odds in e-cigarette users (AOR=4.74; 95% CI: 0.84–26.80; p=0.0784) compared to non-smokers. Poor oral hygiene, defined as visible plaque and calculus on multiple tooth surfaces with gingival inflammation, was the strongest independent factor associated with disease (AOR=38.98; 95% CI: 4.79–317.11; p=0.0012).

**CONCLUSIONS:**

Both cigarette and e-cigarette use were associated with worse periodontal health compared to non-smokers. The elevated odds for e-cigarette users, although accompanied by wide confidence intervals, indicate a potential harmful effect that warrants cautious interpretation. Dental professionals should recognize all forms of tobacco use, including e-cigarettes, as potential risk factors for periodontal disease and emphasize preventive care.

## INTRODUCTION

Oral diseases are among the most widespread health conditions globally, posing significant burdens on individuals’ general health, well-being, and financial stability. They are known to diminish quality of life and often require long-term management. The most encountered oral conditions include dental caries, periodontal disease, tooth loss, and malignancies affecting the lips and oral cavity. Periodontal disease is characterized by the progressive destruction of the supporting soft and hard tissues of the periodontium. This condition arises primarily from an imbalance in the microbial environment and abnormal host immune responses within the gingival and periodontal tissues^1^.

Several factors contribute to the development and progression of periodontal disease, encompassing both patient-related and environmental influences. Common risk factors include tobacco use, particularly smoking, low socioeconomic status, systemic conditions such as diabetes, poor nutritional habits, psychological stress, and aging. Inadequate oral hygiene practices, limited awareness regarding proper dental care, and non-compliance with maintenance regimens further exacerbate periodontal breakdown. These factors, individually or in combination, can disrupt the host response and accelerate the deterioration of periodontal tissues^2^.

Cigarette smoking is widely recognized as a major modifiable risk factor for periodontal disease. Numerous studies have established a direct association between tobacco use and the severity of periodontal destruction, with smokers exhibiting higher rates of clinical attachment loss, pocket depth, and alveolar bone loss compared to non-smokers^3^. The mechanisms underlying this relationship include the deleterious effects of nicotine and other toxicants on immune function, vascular supply, and tissue repair. These substances impair neutrophil function, promote the release of pro-inflammatory cytokines, and reduce blood flow through vasoconstriction, ultimately accelerating disease progression^4^.

In recent years, e-cigarettes have emerged as a popular alternative among individuals seeking to reduce or quit traditional smoking, or as a substitute in environments where smoking is restricted^5^. The global prevalence of e-cigarette use has risen sharply, particularly among youth and young adults. For instance, data from the United States indicate that approximately 14.1% of high school students^6^ and 3.3% of adults were current e-cigarette users as of 2022^7^. In Europe, usage rates vary, but countries such as France, the UK, and Poland have reported increasing trends, especially among young males^8^. In Saudi Arabia, a 2022 study reported that approximately 26% of adults had tried e-cigarettes at least once, with usage notably higher among males aged 18–24 years^9^.

Despite their growing use, concerns have been raised regarding their safety, particularly with respect to oral and periodontal health. E-cigarette aerosols contain a variety of potentially harmful components, including nicotine, heavy metals such as lead, copper, and aluminum, and other cytotoxic compounds^10^. Although marketed as a safer option, the molecular similarities between cigarette smoke and e-cigarette vapor suggest shared biological effects, particularly those related to nicotine-induced vasoconstriction and immune suppression. These effects may compromise gingival blood flow and reduce immune surveillance, thereby increasing susceptibility to periodontal inflammation and tissue destruction^5,11^. Recent studies have also highlighted the oral health impacts of tobacco use and e-cigarettes, including altered self-perceptions and awareness among young adult users^11^, the role of dentists in raising awareness of tobacco’s oral effects and promoting cessation^12^, and the broader effects of various tobacco products on oral health outcomes^13^. Early evidence supports these concerns, indicating that while the severity of damage may differ, e-cigarette use can still negatively impact periodontal health^14-16^.

Although the global and national prevalence of e-cigarette use continues to rise – particularly among youth and young adults – research on its long-term health implications remains limited, especially in relation to oral and periodontal health. While some international studies have begun to explore these associations, there is a noticeable lack of data specific to the Saudi population. To date, only a few studies have addressed the impact of e-smoking on oral health, leaving a clear gap in the literature. In response to this need, the present study aims to evaluate the effects of e-cigarette use on periodontal health among adults in the Eastern Province of Saudi Arabia.

## METHODS

### Study design and setting

This study employed a cross-sectional design to evaluate the impact of e-cigarette use on periodontal health among adults in Saudi Arabia. It was conducted at the undergraduate dental clinics of Imam Abdulrahman Bin Faisal University during the 2025 academic year. Ethical approval was obtained from the Institutional Review Board (IRB) prior to participant enrollment, in accordance with established ethical standards for research involving human subjects^17^.

### Participants and sampling

A total of 169 adult participants, aged 18–65 years, were recruited using a non-probability convenience sampling technique. Individuals were selected from those attending dental care services at Imam Abdulrahman Bin Faisal University during the study period. This method was chosen for its practicality in accessing a diverse clinical population. The inclusion criteria required participants to be dentate, with at least two adjacent teeth, to allow for proper periodontal assessment. Participants who did not meet these inclusion criteria were excluded. This included edentulous individuals, those with pathological oral lesions such as cysts or tumors, and individuals with skeletal abnormalities that could interfere with clinical examination. In addition, patients receiving medications that affect bone metabolism – such as bisphosphonates or RANKL inhibitors – were excluded to ensure reliable periodontal evaluation.

All participants completed a structured questionnaire capturing demographic data, medical history, oral hygiene practices, and self-reported tobacco use, including the type, frequency, and duration of e-cigarette, conventional cigarette, or hookah consumption.

### Data collection procedures

Data collection for this study involved a combination of clinical periodontal examinations and the administration of a structured questionnaire. The clinical component focused on key periodontal parameters, including probing depth (PD), clinical attachment loss (CAL), and gingival recession. These assessments were conducted using a UNC-15 periodontal probe and a standard mouth mirror. All clinical measurements were performed by trained dental professionals who had undergone calibration procedures to ensure consistency and accuracy. Both inter-examiner and intra-examiner reliability were established prior to data collection to ensure validity and reproducibility.

Based on the clinical examination, practitioners assessed and recorded each participant’s oral hygiene status, body mass index (BMI), and periodontal diagnosis. Periodontal disease was diagnosed according to the 2017 World Workshop on the Classification of Periodontal and Peri-Implant Diseases and Conditions^18^. Participants were classified as having periodontal disease if they presented with interdental clinical attachment loss (CAL) of ≥3 mm at two or more non-adjacent teeth, or buccal/oral CAL ≥3 mm with probing depth (PD) ≥3 mm at two or more teeth. This definition was applied to generate a binary outcome (yes, no) for analysis.

BMI was calculated from weight and height measured in the clinic, and the periodontal diagnosis – documenting disease stage and extent – was established using standardized classification criteria. All findings were recorded on a structured clinical assessment form and used for analysis.

Additionally, a structured questionnaire was administered by the practitioners and completed in-person with each participant. While the form was completed by the practitioners, the responses were self-reported by the participants. The questionnaire collected information on demographic characteristics including age, sex, nationality, education level, and monthly income. It also gathered self-reported data on smoking habits (type of tobacco used – e-cigarettes, conventional cigarettes, or hookah – along with frequency and duration), oral hygiene practices such as tooth brushing and flossing, and medical history, including the presence of chronic conditions such as diabetes and hypertension. The questionnaire was adapted from previously validated tools used in periodontal epidemiological research to ensure both reliability and contextual relevance, as demonstrated by Taylor and Borgnakke^19^ in validating self-reported measures for periodontal disease in population studies.

For oral hygiene status, participants were clinically classified into three categories based on the amount of visible plaque, calculus, and gingival inflammation: ‘good’ (minimal plaque, healthy gingiva); ‘fair’ (localized plaque/calculus with mild gingival inflammation); and ‘poor’ (generalized plaque/calculus deposits and gingival inflammation on multiple tooth surfaces).

Smoking frequency was categorized as: ‘heavy’ if participants reported ≥10 cigarettes per day or ≥10 e-cigarette vaping sessions per day; and ‘light’ if they reported less than these amounts. Smoking duration was categorized as: ‘short’ (<3 years) or ‘long’ (≥3 years) based on participant self-report.

These categories were created to ensure adequate group sizes for analysis. All covariates were analyzed as categorical variables in the statistical models, and reference categories were specified in the regression analysis.

### Data management

To ensure data accuracy and minimize entry errors, all collected data were independently entered by two separate investigators into Microsoft Excel. The two datasets were then cross-checked and compared for consistency. After verification and validation, the finalized dataset was imported into SAS software (version 9.4; SAS Institute Inc., Cary, NC, USA) for comprehensive statistical analysis.

### Statistical analysis

Descriptive statistics were applied to summarize all collected variables, including demographic characteristics, tobacco use patterns, and clinical parameters. Categorical variables were reported as frequencies and percentages. Although body mass index (BMI) and income were originally collected as continuous measures, both were recoded into categorical variables for analysis. All continuous variables in this study were categorized using standard clinical or contextual cutoffs to improve interpretability and ensure adequate group sizes for statistical testing. Due to this categorization, no direct statistical tests for differences in continuous variables between groups were performed.

To explore the association between different types of tobacco use (e-cigarettes, conventional cigarettes, hookah) and periodontal status, the Rao–Scott chi-squared test was employed. This test accounts for complex sample design and was used to compare periodontal status across user groups and non-users.

To assess the effect of tobacco, use type on periodontal status, logistic regression analyses were performed. Crude logistic regression models were first used to estimate unadjusted associations. These were followed by multivariable logistic regression models adjusting for potential confounding variables, including age, sex, income level, education level, BMI, systemic health conditions (such as diabetes and hypertension), as well as duration and frequency of tobacco use. Covariates were selected *a priori* based on biological plausibility and evidence from previous literature on periodontal disease risk factors. Variables were retained in the model regardless of statistical significance to ensure adequate control for confounding, and multicollinearity was assessed prior to model fitting.

Associations were reported as odds ratios (ORs) with corresponding 95% confidence intervals (CIs). A p<0.05 was considered statistically significant, and all tests were two-tailed. All analyses were conducted using SAS statistical software, version 9.4 (SAS Institute Inc., Cary, NC, USA), ensuring methodological rigor and reproducibility^10^.

### Sample size and precision considerations

This exploratory clinic-based study did not include an *a priori* power calculation; enrollment was determined by a predefined data-collection period. After data lock, we conducted a *post hoc* sensitivity appraisal to contextualize precision. Given the final sample (n=169) and the observed outcome prevalence (about 67%), the study had adequate sensitivity to detect very large associations (e.g. cigarettes vs non-smokers) but limited sensitivity for moderate associations (e.g. e-cigarettes vs non-smokers). This is reflected in the wide confidence intervals around some estimates. Accordingly, we emphasize effect sizes and their 95% confidence intervals rather than binary significance.

### Ethical considerations

The study protocol was reviewed and approved by the Institutional Review Board (IRB) of Imam Abdulrahman Bin Faisal University prior to the initiation of data collection. Participation in the study was entirely voluntary, and all participants provided written informed consent before enrollment. The consent process included a clear explanation of the study’s objectives, procedures, potential risks, and anticipated benefits to ensure participants were fully informed. All ethical procedures were conducted in accordance with the principles outlined in the Declaration of Helsinki, which governs research involving human subjects^7^.

## RESULTS

A total of 169 adults were included in this cross-sectional study. The majority of participants were male (90.5%) and Saudi nationals (88.8%). Most were aged 18–30 years (62.7%) and reported a monthly income between 5000 and 15000 SAR (60.9%). Nearly half (49.1%) held a high school diploma or less, while 33.7% had a Bachelor’s degree (Supplementary file Table 1). Approximately 89% reported using some form of tobacco, with 37.3% exclusively using e-cigarettes, 27.2% smoking cigarettes, and 18.3% reporting mixed use. Non-smokers comprised 11.8% of the sample. Periodontal disease was diagnosed in 66.9% of participants (Supplementary file Table 2).

**Figure 1:**
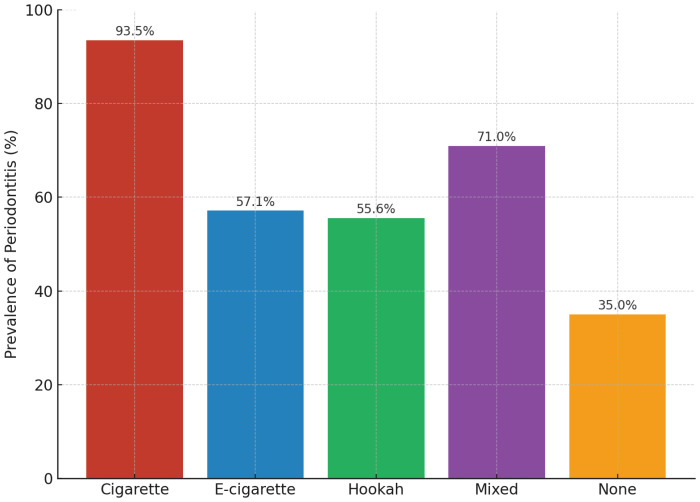
Prevalence of periodontitis according to smoking status, highlighting the higher rates among cigarette and mixed tobacco users compared to non-smokers

Chi-squared analysis revealed significant associations between age, gender, nationality, and medication use with the presence of periodontal disease (Table 1). Significant associations were also observed for oral hygiene status, flossing, brushing frequency, and tobacco-use variables (Table 2). Participants with clinically assessed poor oral hygiene (p<0.0001), infrequent flossing (p=0.001), and brushing less than twice daily (p<0.0001) had a higher prevalence of periodontal disease. Tobacco type was likewise significantly associated with periodontal disease (p<0.0001). As illustrated in Figure 1, cigarette and mixed tobacco users had the highest prevalence rates (93.5% and 71.0%, respectively), followed by e-cigarette users (57.1%) and hookah users (55.6%), while non-smokers had the lowest prevalence (35.0%).

**Table 1:** Demographic factors associated with the presence of periodontitis (N=169)

	*Total* *n*	*With* *periodontitis* *n*	*Without* *periodontitis* *n*	p*
**Age** (years)				**<0.0001**
18–30	106	54	52	
31–40	31	28	3	
≥41	32	31	1	
**Gender**				**0.0087**
Male	153	107	46	
Female	16	6	10	
**Nationality**				**0.0061**
Saudis	150	95	55	
Non-Saudis	19	18	1	
**Income** (SAR)				**0.2701**
5000–15000	103	70	33	
<5000	40	29	11	
>15000	26	14	12	
**Systemic diseases**				0.1306
Diseased	25	20	5	
None	144	93	51	
**Medication**				0.0022
No	152	96	56	
Yes	17	17	0	
**Education level**				**0.0110**
Bachelor’s	57	31	26	
Diploma	29	25	4	
≤High school	83	57	26	
**BMI**				0.8143
Normal	83	55	28	
Obese	21	13	8	
Overweight	65	45	20	

* Two-tailed Rao–Scott chi-squared tests. SAR: 1000 Saudi Riyals about US$270.

**Table 2:** Tobacco-use patterns and oral hygiene behaviors associated with the presence of periodontitis (N=169)

	*Total* *n*	*With* *periodontitis* *n*	*Without* *periodontitis* *n*	p*
**Smoking**				0.0013
Yes	106	106	43	
No	31	7	13	
**Smoking group**				<0.0001
Cigarette	46	43	3	
E-cigarette	63	36	27	
Hookah	9	5	4	
Mixed	31	22	9	
None	20	7	13	
**Smoking frequency**				<0.0001
Heavy	50	44	6	
Light	31	23	8	
None	88	46	42	
**Smoking duration (years)**				<0.0001
Long >3	104	82	22	
Short ≤3	45	24	21	
None	20	7	13	
**Oral hygiene**				<0.0001
Poor	42	41	1	
Fair	69	52	17	
Good	58	20	38	
**Brushing frequency (per day)**				<0.0001
≥2	81	39	42	
1	68	56	12	
Doesn’t brush	20	18	2	
**Flossing group**				0.0010
No	131	96	35	
Yes	38	17	21	

* Two-tailed Rao–Scott chi-squared tests.

**Figure 2:**
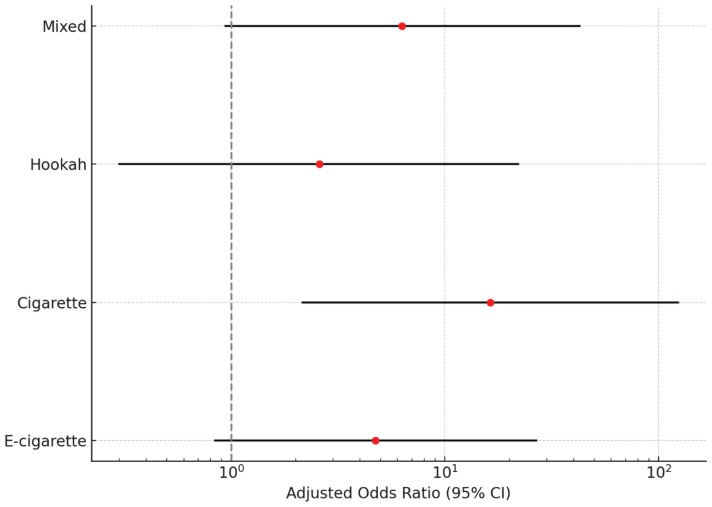
Adjusted odds ratios (95% CI) for periodontitis by tobacco type from multivariable logistic regression, showing the substantially increased risk among cigarette smokers and mixed tobacco users compared to non-smokers

In the crude logistic regression model, e-cigarette users had higher odds of periodontal disease compared to non-smokers (OR=2.48; 95% CI: 0.85–7.21; p=0.0959). Cigarette users had markedly elevated odds (OR=26.62; 95% CI: 5.81–121.87; p<0.0001). In the adjusted model controlling for age, BMI, education, hygiene, and systemic health, the association for e-cigarettes remained elevated (AOR=4.74; 95% CI: 0.84–26.80; p=0.0784), while cigarette use continued to show a strong association (AOR=16.31; p=0.0071). These adjusted estimates are visualized in Figure 2, which demonstrates the substantially higher odds of periodontitis among cigarette smokers and mixed tobacco users compared to non-smokers. No difference in periodontal disease odds was observed between e-cigarette and cigarette users (Table 3).

**Table 3:** Logistic regression analysis for the association between different types of smoking and periodontitis in adults

*Tabaco use*	*OR (95% CI)*	*p*	*AOR (95% CI)*	*p*
Non-smoker (ref.)	1		-	
E-cigarette	2.48 (0.85–7.21)	0.0959	4.74 (0.84–26.80)	0.0784
Cigarette	26.61 (5.81–121.87)	<0.0001	16.31 (2.16–123.18)	0.0071
Hookah	2.32 (0.45–11.97)	0.3122	2.58 (0.30–21.99)	0.3844
Mixed	4.54 (1.33–15.52)	0.0162	6.30 (0.94–42.63)	0.0579

AOR: adjusted odds ratio obtained from a multivariable logistic regression model controlling for BMI category, age group, gender, nationality, education level, oral hygiene, flossing frequency, and presence of systemic conditions.

* p<0.05.

Poor oral hygiene was the most robust independent factor associated with disease: individuals with poor hygiene had nearly 40 times the odds of disease (p=0.0010), and those with fair hygiene also showed increased risk (OR=4.21; p=0.0194). Other covariates were not significantly associated in the adjusted model.

## DISCUSSION

This cross-sectional study examined the association between e-cigarette use and periodontal disease among Saudi adults. The findings revealed that both e-cigarette and cigarette users had higher rates of periodontal disease compared to non-smokers. While the association for cigarette use was statistically significant and that for e-cigarette use showed elevated odds with wider confidence intervals, the lack of precision should not be interpreted as the absence of risk. Instead, it may reflect the limited sample sizes in some subgroups, which could have reduced the ability to detect potential differences. Further studies with larger, more representative samples are needed to better clarify the relationship between e-cigarette use and periodontal health.

Our findings are consistent with previous research indicating that e-cigarette use can negatively affect periodontal tissues, though the extent may be less pronounced than with traditional tobacco smoking^18,20^. Prior studies have shown that e-cigarette aerosols may trigger inflammatory responses, compromise immune function, and disrupt the oral microbiome – factors known to contribute to periodontal breakdown^17,21,22^. In addition to these pathways, e-cigarette aerosols have been shown to induce oxidative stress through the generation of reactive oxygen species, which can impair cellular repair processes and exacerbate periodontal tissue damage. Alterations in the oral microbiome associated with e-cigarette use include increased colonization by pathogenic bacteria and reduced microbial diversity, both of which can heighten inflammatory responses. Furthermore, trace heavy metals such as lead, nickel, and chromium – detected in e-cigarette vapor – pose potential cytotoxic and pro-inflammatory effects on gingival and periodontal tissues^10,11,23^. These mechanistic insights provide biological plausibility for the observed associations between e-cigarette use and poorer periodontal health outcomes^24-26^, even in the absence of statistically significant findings in our study.

Furthermore, Pushalkar et al.^27^ demonstrated that e-cigarette aerosol alters the composition of the oral microbiome and increases susceptibility to infection, supporting the potential mechanistic basis for periodontal disease progression among users. Additionally, self-reported data from international surveys among dental students have highlighted common adverse oral health outcomes associated with e-cigarette use, including gingival irritation and dryness, further validating our findings from a user-experience perspective^28^.

Moreover, participants who reported using both e-cigarettes and conventional tobacco products demonstrated the most severe periodontal involvement, suggesting a possible cumulative or synergistic effect. This aligns with previous evidence that dual use is associated with elevated oral health risks^19,20,24^. Beyond local periodontal effects, emerging evidence has also raised concerns regarding the systemic and carcinogenic risks linked to chronic e-cigarette use. These considerations reinforce the importance of viewing e-cigarette use as a public health concern, not only from a periodontal but also from a broader systemic health perspective.

In addition to tobacco exposure, poor oral hygiene and increasing age emerged as significant factors associated with periodontal disease in our multivariable model, reinforcing the multifactorial nature of periodontal pathology^8^. While e-cigarettes are often marketed as a safer alternative to combustible tobacco, our data highlight the need for greater public health awareness and professional caution regarding their potential oral health risks. As the prevalence of e-cigarette use continues to rise, especially among young adults, there is an urgent need for further longitudinal research to clarify the long-term effects of these products on periodontal and systemic health^25,26^.

### Strengths and limitations

The study’s strengths include a clearly defined adult population, standardized clinical assessments, and the use of adjusted statistical models to control for confounding. However, several limitations should be considered. The cross-sectional design precludes causal inference. The relatively small sample – especially in some subgroups – may have limited statistical power, meaning the study was better equipped to detect very large associations than moderate ones; thus, the lack of statistical significance for some findings (e.g. e-cigarette use) may reflect limited power rather than absence of risk. For e-cigarette use specifically, the wide confidence intervals crossing unity limit the precision of the estimate and warrant cautious interpretation. The sample was predominantly male (90.5%), and the single-center setting, combined with the use of a non-probability convenience sampling method, may limit generalizability to other populations and care contexts and introduce potential selection bias. ‘Mixed smokers’ were defined as users of more than one tobacco product, but predominant product use was not captured, limiting sensitivity analyses and introducing potential misclassification. Finally, self-reported smoking behavior may be subject to recall or social-desirability bias, and residual confounding remains possible given that certain variables – such as diet, alcohol use, stress levels, and genetic predisposition – were not measured. Future longitudinal studies with larger, more diverse, multi-center samples using probability-based sampling methods are warranted.

## CONCLUSIONS

This study found that e-cigarette use was associated with a higher prevalence of periodontal disease compared to non-smokers, with elevated odds and wide confidence intervals. The absence of statistical significance in this association may be due to sample size limitations rather than the absence of risk. In contrast, conventional cigarette smoking demonstrated a significant association with increased periodontal disease prevalence. Additionally, hookah use and mixed tobacco use were also associated with substantial increases in periodontal disease prevalence, underscoring that all forms of tobacco pose risks to periodontal health. These findings highlight the need to include all tobacco products – including e-cigarettes – within dental risk assessments and preventive care strategies. Further longitudinal studies with larger and more diverse populations are warranted to clarify the long-term impact of each tobacco type on periodontal tissues.

## Supplementary Material



## Data Availability

The data supporting this research are available from the authors on reasonable request.
