# Supplementary material for: The effect of cigarette and e-cigarette use on periodontal health: A cross-sectional study in Eastern Province, Saudi Arabia
Source: Tob Induc Dis. 2026 Jan 17;24:10.18332/tid/209573. doi: 10.18332/tid/209573 (PMC12811842; doi:10.18332/tid/209573)
Supplement: Supplementary file 1 [file TID-24-04-s1.pdf]

Supplementary Table 1. Demographic Characteristics of Study Participants.

	**Total (n)**	**Percentage %**
**Age (N = 169)**		
18-30	106	62.72
31-40	31	18.34
41+	32	18.93
**Gender (N = 169)**		
Male	153	90.53
Female	16	9.47
**nationality (N = 169)**		
Saudis	150	88.76
Non- Saudis	19	11.24
**Income (N = 169)**		
5000-15000	103	60.95
<5000	40	23.67
>15000	26	15.38
**Systemic diseases (N = 169)**		
None	144	85.21
Diseased	25	14.79
**Medication (N = 169)**		
No	152	89.94
Yes	17	10.06
**Education (N = 169)**		
Bachelor's	57	33.73
Diploma	29	17.16
≤High School	83	49.11
**BMI (N = 169)**		
Healthy	83	48.52
Obese	21	12.43
Overweight	65	38.46

Supplementary Table 2. Smoking and dental health habits of Study Participants

	**Total (n)**	**Percentage %**
**Smoking (N = 169)**		
Yes	149	88.17
No	20	11.83
**Smoking group (N = 169)**		
Cigarette	46	27.22
E-Cigarette	63	37.28
Hooka	9	5.33
Mixed	31	18.34
None	20	11.83

**Smoking Frequency (N = 169)**		
Heavy	50	29.59
Light	31	18.34
None	88	52.07
**Smoking Duration (N = 169)**		
Long >3 years	104	61.54
Short ≤ 3	45	26.63
None	20	11.83
**Oral hygiene (N = 169)**		
Poor	42	24.85
Fair	69	40.83
Good	58	34.32
**Brushing frequency (N = 169)**		
≥ Twice/Day	81	47.93
Once/day	68	40.24
Doesn't brush	20	11.83
**Flossing group (N=169)**		
No	131	77.51
Yes	38	22.49
**Periodontal disease (N = 169)**		
No	56	33.14
Yes	113	66.86

© 2026 Aljoghaiman E.A. et al.
